# Mitochondria and Parkinson's Disease

**DOI:** 10.4061/2011/261791

**Published:** 2012-08-29

**Authors:** David K. Simon, Charleen T. Chu, Russell H. Swerdlow

**Affiliations:** ^1^Beth Israel Deaconess Medical Center and Harvard Medical School, 330 Brookline Avenue, Room CLS-638, Boston, MA 02215, USA; ^2^Department of Pathology, University of Pittsburgh School of Medicine, Pittsburgh, PA 15261, USA; ^3^Departments of Neurology, Physiology, and Biochemistry and Molecular Biology and University of Kansas Alzheimer's Disease Center, University of Kansas Medical Center, 3901 Rainbow Boulevard, Kansas City, KS 66160, USA

A large body of evidence implicates a central role for mitochondrial dysfunction in the pathogenesis of Parkinson's disease (PD), although the precise causes of mitochondrial dysfunction in PD remain to be determined. Mitochondrial complex I activity is impaired in the substantia nigra at early stages in PD. This complex I dysfunction appears not to be simply a consequence of neurodegeneration as toxins that inhibit mitochondrial complex I can reproduce many of the key pathological features of PD in animal models. Several genetic causes of PD have been linked directly or indirectly to mitochondrial function, and thus a convergence of data highlights the role of mitochondrial dysfunction in PD. This special issue now explores several key issues relating to the role of mitochondrial dysfunction in PD, potential causes and consequences of the dysfunction, and implications regarding neuroprotective strategies.

The first paper by A. H. V. Schapira and M. Gegg provides a brief but broad overview of mitochondrial dysfunction in PD. In addition to a discussion of parkinsonism associated with mutations in mitochondrial DNA (mtDNA) polymerase gamma (POLG), links to abnormal mitochondrial dysfunction are reported for several genetic causes of PD, including PINK1, Parkin, DJ-1, alpha-synuclein, and HtrA2. The role of PINK1, Parkin, and DJ-1 in the degradation of dysfunctional mitochondria (mitophagy) is discussed also.

The second paper by J. Clark, Y. Dai, and D. K. Simon reviews data regarding the potential contribution of mtDNA mutations to PD. This review briefly discusses evidence for mitochondrial dysfunction in PD, as well as data from cytoplasmic hybrid (“cybrid”) cell lines that implicate a role for mtDNA mutations as a cause of the mitochondrial dysfunction. The paper then focuses primarily on data suggesting a role for somatic mtDNA mutations. The significance to PD of data from the POLG “mutator” mouse model of premature aging also is discussed, including the issue of point mutations versus large deletions.

The third article by C. T. Chu reviews the evidence for altered autophagy or mitophagy in the major genetic models of parkinsonian neurodegeneration, *α*-synuclein, leucine-rich repeat kinase 2 (LRRK2), Parkin, PTEN-induced kinase 1 (PINK1), and DJ-1. In recent years, autophagy dysregulation has been implicated widely in toxic/environmental and genetic approaches to studying PD pathogenesis. Diminished autolysosomal surveillance leads to alterations in mitochondrial oxygen utilization or calcium buffering. Interactions of two recessive PD-linked proteins, PINK1 and Parkin, contribute to a mechanism by which dissipation of the inner mitochondrial membrane potential triggers selective mitochondrial targeting for autophagy. These data are discussed in the context of emerging knowledge in the basic regulation of autophagy induction and cargo targeting, with discussion of neuron-specific mechanisms that may differ from those observed in other cell types.

The fourth article by A. Rakovic et al. is a research article in which tandem affinity purification and mass spectrometry were utilized to confirm interactions of PINK1 with HSP90 and CDC37 molecular chaperones, while highlighting 4 mitochondrially localized proteins that may interact with PINK1. Although no changes in protein expression were observed in control versus PINK1 mutant fibroblast lines, mutations in the *LRPPRC* gene encoding one of these proteins cause a form of Leigh syndrome, a cytochrome c oxidase deficiency that results in childhood brain infarcts. Further studies of PINK1 regulation of mitochondrial function may well reveal functions beyond those linked to Parkin-regulated mitophagy.

The fifth paper by P. C. Keane et al. reviews mechanisms through which mitochondrial dysfunction may impair function and induce neurodegeneration in PD, as well as mediate the adverse consequences of gene mutations known to associate with Mendelian PD. Relationships between mitochondria and oxidative stress, cell calcium homeostasis, dopamine metabolism, and Lewy bodies are addressed. What we have learned over the years regarding PD-associated toxins, as well as mutations in genes such as Parkin, Pink1, alpha synuclein, and DJ1, are discussed from the perspective of mitochondria and cell bioenergetics. Overall, this review makes a compelling case for why mitochondria should be considered to play a key role in both PD etiology and physiology.

The sixth paper, a review by K. U. Tufekci et al., focuses on compensatory transcriptional responses to oxidative stress mediated by the Nrf2 transcription factor. Through the electrophile response element, Nrf2 participates in upregulation of antioxidant and anti-inflammatory genes and affects mitochondrial biogenesis. Interestingly, the survival-death outcome in injuries associated with increased mitophagy may be governed by whether or not there are concurrent deficiencies in mitochondrial biogenesis, as observed in a chronic culture model of MPP+ intoxication (Zhu et al., *Cell Death and Disease,* 2012 May 24; 3:e312. doi: 10.1038/cddis.2012.46). Given recent data implicating crosstalk between autophagy and Nrf2, Nrf2 may represent a promising therapeutic target addressing several mechanisms implicated in PD pathogenesis.

In the seventh paper, D. M. Arduíno et al. address the fact that, in addition to mitophagy, other aspects of mitochondrial dynamics have been implicated in PD models. These authors review recent studies implicating changes in mitochondrial fission and fusion, mitochondrial biogenesis, degradation, and transport along microtubules in the major genetic models of PD. A model whereby deficits in mitochondrial bioenergetics may underlie each of these changes is proposed, although several PD genes may also show direct effects on mitochondrial transport or mitochondrial fission.

The eighth paper by A. R. Esteves et al. provides a broad review of evidence for mitochondrial dysfunction in PD as well as potential causes and consequences of this dysfunction. The topics include oxidative stress, calcium homeostasis, the NAD+/NADH ratio, autophagy, and mitophagy. The “mitochondrial cascade” hypothesis is discussed in which each of these mechanisms ultimately contributes to alpha-synuclein aggregation followed by neuronal death.

Finally, the ninth paper is a review by R. B. Mounsey and P. Teismann that provides a historical overview of data implicating mitochondria in PD pathogenesis. This paper spans early data from idiopathic PD through consideration of the MPTP, rotenone and 6-hydroxydopamine models, to recent work exploring how genetic mutations influence mitochondrial activity. The preclinical data underlying several potential mitochondrially targeted therapies are discussed.

*David K. Simon* *David K. Simon* *Charleen T. Chu* *Charleen T. Chu* *Russell H. Swerdlow* *Russell H. Swerdlow*

